# Supplementary material for: Avatar error in your favor: Embodied avatars can fix users’ mistakes without them noticing
Source: PLoS One. 2023 Jan 20;18(1):e0266212. doi: 10.1371/journal.pone.0266212 (PMC9858437; doi:10.1371/journal.pone.0266212)
Supplement: S1 Appendix — An appendix detailing the demographic questionnaire, the used inverse kinematics, and the finger swap animation is provided as a separate document. (PDF) [file pone.0266212.s001.pdf]

1 Demographic questionnaire

Question	Possible answers
Gender	Male<br>Female<br>Other<br>I don't want to answer
Age	Integer
Height	Integer (centimeters)
Weight	Integer (kilograms)
Handedness	Right handed<br>Left handed<br>Ambidextrous
Main occupation	Text
Have you ever experienced "virtual reality" before?	Linear scale between 0 (No experience) and 7 (Daily use)
Do you practice sport?	Linear scale between 0 (No) and 7 (Daily practice)
Do you often type (piano/keyboards/etc.)?	Linear scale between 0 (No) and 7 (A lot)

Inverse Kinematics

Both arms and fingers IKs relies on the same principle: with known bones lengths (through a calibration process) and mechanical constraints (single joint for the elbow, the same flexion rotation for both proximal-intermediate and intermediate-distal joints [1]), we can determine joints rotations in the plan (the elbow or finger joints can only bend alongside a single axis). The final hand animation step is to realign the fingertips with their associated marker as described in [2]. This additional rotation follows a predefined curve based on the lateral position of the hand was added alongside the elbow-wrist axis (the elbow is not constantly stuck near the ribs when the target is within range). Finally, half of the swivel angle of the wrist is applied to the elbow joint to avoid mesh rigging issues.

Finger swap animation

When a swap is introduced, only the vertical motion of the *real source finger* (the finger the subject moves) is redirected onto the *displayed destination finger* (the finger the subject sees moving) to avoid potential lateral interpenetration.

At the beginning of the swap, the fingers' markers' positions are stored. Then, the lateral motion of the *displayed source finger*'s (i.e., what the users see for the source finger) motion is progressively shifted back to its initial position while the vertical motions used to animate both displayed fingers are progressively permuted. A custom anatomical correction is applied to scale the measured vertical movement to the size of the moving finger (e.g., when swapping the middle finger with the little finger).

The progressive removal of the lateral motion prevents the user from visually spotting the source finger while the swap is enabled and the lateral motion of the destination finger is not altered. Thus, if the subject moves the *real destination finger*

(i.e., the finger initially not supposed to be moved by the user but still animated by the swap) laterally while the swap is active, the *displayed destination finger* will also move laterally, but its vertical motion will be the one of the *real source finger*. Conversely, if the user moves the *real source finger* laterally, nothing will move on screens, and if it moves vertically only the *displayed destination finger* will move.

The displayed fingers' positions are computed using Equation 1 with :

- t refers to the rate of swap introduced and varies from 0 (motions are still fully congruent) to 1 (motions are fully permuted) with a step set to 0.4 per frame ($\simeq 31ms$) for the activation and 0.02 for the release ($\simeq 625ms$).
- $\vec{P}_{\mathrm{xz}_{\mathrm{ref}}}$ is the planar position of the *real source finger* tip when the swap is triggered
- $\vec{P}_{\mathrm{xz}}$ is the planar position of the *real source finger* tip and $\vec{P}_{\mathrm{xz}_d}$ the one for the *displayed source finger*
- h_{src} is the vertical component of the *real source finger* tip position and h_{dst} the vertical component of the *real destination finger* tip, with h_{src_d} and h_{dst_d} the *displayed* ones respectively

$$\begin{cases} \vec{P}_{\mathrm{xz}_d} &= (1-t)\cdot\vec{P}_{\mathrm{xz}} + t\cdot\vec{P}_{\mathrm{xz}_{\mathrm{ref}}} \\ h_{\mathrm{src}_d} &= (1-t)\cdot h_{\mathrm{src}} + t\cdot h_{\mathrm{dst}} \\ h_{\mathrm{dst}_d} &= (1-t)\cdot h_{\mathrm{dst}} + t\cdot h_{\mathrm{src}} \end{cases} \tag{1}$$

The swap ends when the moving finger is placed back on the table or when the current button leaves the activation area. Such a method does not introduce any additional delay in the fingers' animation when a swap is introduced.

References

1. Aristidou A. Hand tracking with physiological constraints. The Visual Computer. 2018;34(2):213–228. doi:10.1007/s00371-016-1327-8.
2. Pavllo D, Delahaye M, Porssut T, Herbelin B, Boulic R. Real-time neural network prediction for handling two-hands mutual occlusions. Computers & Graphics: X. 2019;2:100011. doi:https://doi.org/10.1016/j.cagx.2019.100011.
